# Impact of breakfast consumption timing *v*. breakfast omission on post-lunch glycaemia and insulinaemia in adolescent girls: a randomised crossover trial

**DOI:** 10.1017/S0007114525000248

**Published:** 2025-03-14

**Authors:** Sahar Afeef, Julia K. Zakrzewski-Fruer, Alice E. Thackray, Laura A. Barrett, Keith Tolfrey

**Affiliations:** 1 Department of Clinical Nutrition, Faculty of Applied Medical Sciences, King Abdulaziz University, Jeddah, Saudi Arabia; 2 School of Sport, Exercise and Health Sciences, Loughborough University, Loughborough LE11 3TU, UK; 3 Institute for Sport and Physical Activity Research, University of Bedfordshire, Bedford MK41 9EA, UK; 4 NIHR Leicester Biomedical Research Centre, University Hospitals of Leicester NHS Trust and University of Leicester, Leicester, UK

**Keywords:** Glucose, Insulin, Postprandial, Second-meal effect

## Abstract

Adolescent girls often skip breakfast due to time constraints and reduced morning appetite. This study examined the acute impact of breakfast consumption timing *v*. breakfast omission (BO) on glycaemic and insulinaemic responses to lunch in infrequent breakfast-consuming girls. Fifteen girls (13·1 (sd 0·8) years) completed three conditions in a randomised crossover design: early-morning breakfast consumption (EM-BC; 08.30), mid-morning breakfast consumption (MM-BC; 10.30) and BO. A standardised lunch was provided at 12.30, followed by a 2-h post-lunch observation period. Blood and expired gas samples were collected periodically. Linear mixed models with Cohen’s *d* effect sizes compared outcomes between conditions. Pre-lunch glucose and insulin incremental AUC (iAUC) were higher in the breakfast conditions *v*. BO (*P* ≤ 0·009), with no differences between breakfast conditions. MM-BC reduced post-lunch glucose iAUC by 36 % and 25 % compared with BO and EM-BC, respectively (*P* < 0·001, *d* = 0·92–1·44). A moderate, non-significant 15 % reduction in post-lunch glucose iAUC was seen with EM-BC *v*. BO (*P* = 0·077, *d* = 0·52). These reductions occurred without changes in post-lunch insulinemia (*P* ≥ 0·323) and were accompanied by increased post-lunch carbohydrate oxidation compared with BO (*P* ≤ 0·018, *d* = 0·58–0·75); with no differences between EM-BC and MM-BC. MM-BC lowered glycaemic response over the experimental period compared with BO (*P* = 0·033, *d* = 0·98) and EM-BC (*P* = 0·123, *d* = 0·93), with no difference between EM-BC and BO. Compared with BO, both breakfast conditions lowered post-lunch glycaemic responses with mid-morning breakfast eliciting a greater second-meal effect than early-morning breakfast. These findings indicate the breakfast-to-lunch meal interval may be a crucial factor affecting postprandial glycaemia in infrequent breakfast-consuming girls.

Elevated postprandial glycaemia has been implicated in cardiometabolic diseases by promoting oxidative stress, inflammation and endothelial dysfunction^(1,2)^. Even in healthy individuals, postprandial hyperglycaemia is recognised as a significant risk factor for type 2 diabetes (T2D)^(3)^ and CVD^(4)^ development. The prevalence of T2D among young people aged < 17 years is on a concerning rise in the UK^(5)^. Indeed, the risk of T2D increases during puberty^(6)^, partly due to the physiological reduction in insulin sensitivity^(7)^, which if combined with other risk factors such as an unhealthy diet^(8)^, can pose challenges on glycaemic control. Therefore, moderating postprandial glycaemia through dietary interventions is vital for disease prevention^(9)^.

Breakfast consumption is considered an integral component of healthy eating habits. Observational studies demonstrate that frequent breakfast consumption was associated with lower fasting blood glucose and insulin concentrations in adolescents^(10,11)^ and a lower incidence of T2D in adults^(12,13)^. Supporting this association, acute intervention studies with adults show that breakfast consumption reduced postprandial glycaemia, with or without changes in insulin concentrations, in response to a second meal (i.e. lunch) compared with breakfast omission (BO)^(14,15)^. This improvement has been referred to as the ‘second meal effect’ and has been studied mainly in adults. Very little information is available regarding the second-meal effect in adolescents. In this regard, a recent study demonstrated that breakfast consumption significantly reduced glycaemic and insulinaemic responses to lunch, while also increasing carbohydrate oxidation, when compared with BO in adolescent girls who habitually consume breakfast^(16)^. Such findings may not apply directly to adolescent girls classified as infrequent breakfast consumers because previous studies in adults suggest that habitual breakfast skippers may have a less pronounced second-meal effect than habitual breakfast consumers^(17)^. In young women (mean age of 19 years) who habitually skipped breakfast, consuming breakfast of different protein contents did not change the glycaemic and insulinaemic responses to lunch when compared with BO^(18)^. However, these observations were derived from combined data of both adolescents and adults aged 13–20 years, making it difficult to isolate the unique response of adolescents who have different hormonal and metabolic profiles^(19)^. While existing evidence is largely based on adults, this study focussed on examining the impact of breakfast skipping on glycaemic and insulinaemic responses in adolescent girls who infrequently consume breakfast. This is particularly important given the increased prevalence of breakfast skipping during adolescence, specifically among girls (∼20 %)^(20,21)^.

Definitions of ‘breakfast’ provide recommendations on quantity, composition and timing^(22–24)^, yet the latter has not been investigated among adolescents with little data on adults. Timing-wise definitions have suggested that breakfast should be eaten within 2–3 h of awakening^(23)^, often no later than 10.00^(24)^. Nevertheless, conflicting definitions have been utilised across the literature, primarily due to a lack of data supporting an optimal time to consume breakfast. Common reasons for skipping breakfast in adolescent girls are lack of time and not being hungry in the morning^(25)^. Thus, exploring different breakfast timings, such as consuming breakfast later in the morning (10.30) during school breaks, could be an attractive option to facilitate frequent breakfast consumption. Research comparing different breakfast timings would also be valuable in informing the time-of-day aspect of an evidence-based definition of breakfast consumption for research and practical application. A recent study in adult men with overweight or obesity showed that delayed breakfast consumed at 10.00 with lunch consumed in closer proximity (3 h) resulted in lower glycaemic and insulinaemic responses to lunch compared with breakfast consumed earlier in the morning at 07.00 and lunch 5 h later^(26)^. Yet, such responses have not been examined in adolescents, and the effect of breakfast consumption at different times compared to BO is still not known. Hence, the primary aim of this study was to compare the effects of early-morning breakfast consumption (EM-BC, 08.30) *v*. mid-morning breakfast consumption (MM-BC, 10.30) breakfast consumption with BO on post-lunch glycaemia and insulinaemia in adolescent girls classified as infrequent breakfast consumers. The secondary aim was to examine the effect of breakfast timing *v*. BO on substrate oxidation since skipping breakfast is associated with higher postprandial insulinaemia and increased fat oxidation, which represent markers of metabolic inflexibility due to extended fasting^(27)^. Understanding these changes is crucial, as they could impair glucose regulation over time, impacting metabolic health.

## Methods

### Participants

**Figure 1.**
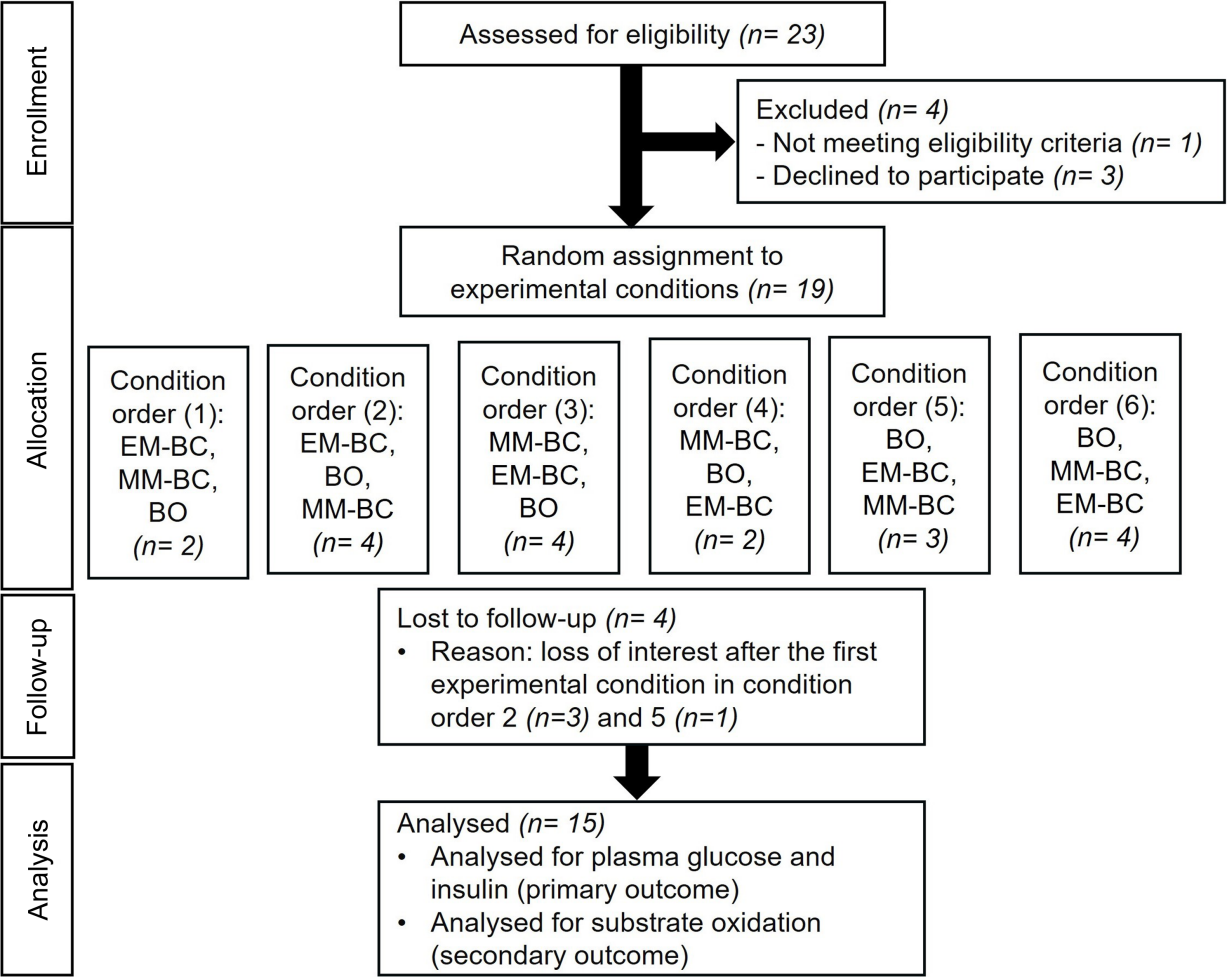
Participant flow chart of adolescent girls who participated in a randomised crossover study design involving three conditions.

After gaining Loughborough University Ethics approval (HPSC reference No. 3129), twenty-three healthy adolescent girls from local secondary schools volunteered to participate in the study. Four participants were excluded from the study during the preliminary visit and another four participants dropped out after the first breakfast condition due to a lack of interest. Therefore, a total of fifteen apparently healthy adolescent girls completed the study, and their data were carried forward for analysis (Figure 1). Data collection took place from November 2021 to July 2022. Participants were screened for general health using a questionnaire and gave their assent, and their caregiver provided written informed consent. Girls were included if they were 11–14 years old, generally healthy, were not allergic to the breakfast ingredients or taking prescribed medicines, were free from any metabolic diseases and were classified as infrequent breakfast consumers during the preliminary visit. The study was registered at clinicaltrials.gov (identifier NCT05000944).

### Preliminary visit

Participants arrived at the research lab after an overnight fast. Stature was measured using a stadiometer (The Leicester height measure, Seca Ltd., Birmingham, UK) to the nearest 0·01 m. Body mass was measured, and percentage body fat was estimated using bioelectrical impedance (Tanita BC-418MA, Tokyo, Japan), while participants stood barefoot wearing light clothes, to the nearest 0·1 kg and 0·1 %, respectively. BMI was calculated by dividing body mass (kg) by stature squared (m^2^). Consequently, weight status was determined using age and sex-specific BMI cut-off points^(28)^. Waist circumference was measured midway between the tenth rib and the iliac crest^(29)^. Subsequently, participants sat quietly for 10 min before being fitted with a suitably sized facemask (Hans Rudolph Inc., Shawnee, USA) to determine the RMR as recommended^(30)^. Gas samples were then collected continuously for 10 min using a breath-by-breath gas analysis system (Vyntus CPX, CareFusion, Germany). The oxygen consumption and carbon dioxide production data were averaged into 5-second intervals and screened for steady-state conditions, with values exceeding a respiratory exchange ratio of 1·00 being excluded, consistent with indirect calorimetry assumptions^(31)^. Energy expenditure was estimated using the equations by Frayn (1983)^(31)^ and the RMR that represented the lowest 5 min rolling average was used to reflect minimum individual energy needs. Participants completed a breakfast habits questionnaire, which included questions about breakfast consumption frequency (days per week), timing and location, foods/drinks choices and reasons for skipping breakfast^(32)^. This study targeted adolescent girls classified as ‘infrequent breakfast consumers’, defined as those who consumed breakfast (> 50 kcal before 10.30) on ≤ 4 days per week. Volunteers who consumed breakfast frequently (i.e. 5–7 days per week) were excluded. The focus on infrequent consumers was based on previous findings that link infrequent breakfast consumption with poorer cardiometabolic health among adolescents^(33–35)^. Participants tasted both breakfast and lunch meals to ensure acceptability. All participants reported finding the meals acceptable. Lastly, the participants were asked to self-assess their physical maturation at home with the assistance of a caregiver using validated secondary sexual characteristics scales^(36,37)^.

### Experimental design

**Figure 2.**
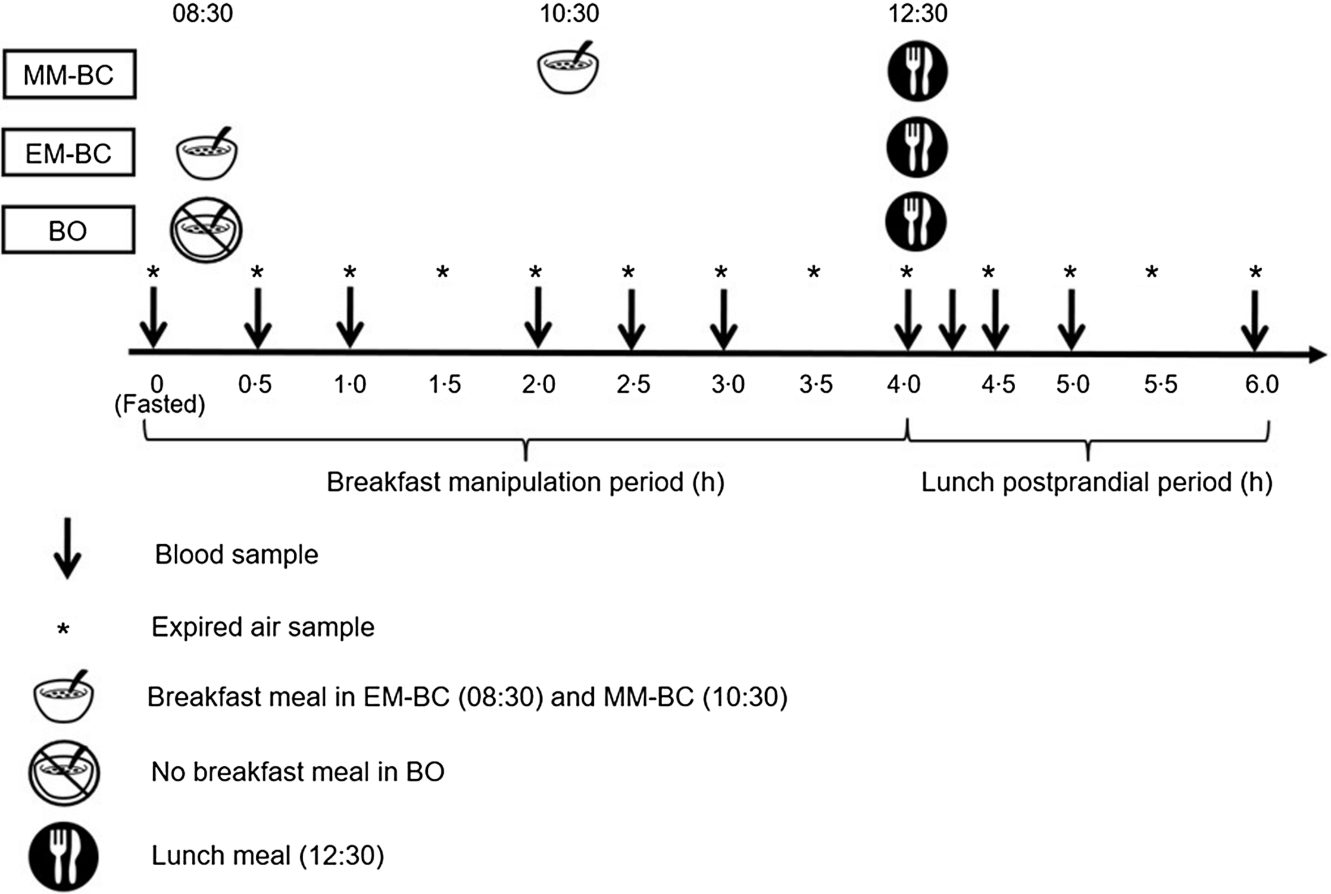
Schematic of the three experimental conditions. BO, breakfast omission; EM-BC, early-morning breakfast consumption; MM-BC, mid-morning breakfast consumption.

This study employed a randomised crossover design. Participants completed three conditions assigned according to the Latin square method: EM-BC, MM-BC and BO. The allocation sequence was generated, and participants were enrolled and assigned to conditions by the first author, SA. The participants attended all sessions in friendship pairs, receiving the same treatment in the same order to maintain consistency across sessions and increase comfort and adherence. Initial attempts were made to control for the menstrual cycle phase, but due to study logistics (friendship pairs), other parental and participant commitments, irregular cycles and availability of research assistants and lab resources, this was not feasible. There was a 3-to-30-day washout period between conditions, with a longer washout period for those with regular menstrual cycles. The participants were unaware of the order of study visits until they were informed on arrival. The participants were asked to record their dietary intake using a weighed food diary for 48 h preceding the first condition and were asked to replicate their intakes for the subsequent conditions. In addition, participants were asked to minimise moderate-vigorous intensity physical activity 48 h before each condition to control for any residual effect on glycaemia and insulinaemia^(38,39)^, which was confirmed via accelerometry. A schematic representation of the study design is provided in Figure 2. The experimental conditions started after an overnight fast and a 10-min rest at 08.00. Expired gas samples were collected with participants seated for 5 min at 0 h (baseline) and every 0·5 h thereafter using a standard Douglas bag method. Participants remained seated throughout the experimental period (08.00–14.30) but used the mouthpiece and nose clip only during the gas collection intervals. Substrate oxidation was estimated using recognised stoichiometric equations^(31)^. Capillary blood samples for the measurement of plasma glucose and insulin were also taken at 0, 0·5, 1, 2, 2·5, 3 and 4 h in the pre-lunch period and at 0·25, 0·5, 1 and 2 h post-lunch. The standardised breakfast was provided immediately after baseline measures at 08.30 for EM-BC and 2 h later at 10.30 for the separate MM-BC condition, with no breakfast being provided for the BO condition. The standardised lunch was consumed at 12.30 for all conditions. *Ad libitum* water intake was allowed during the first experimental condition, with the quantity and timings replicated in the subsequent conditions.

### Standardised test meals

The quantity, composition and timing of the breakfast were in accordance with the proposed definitions of ‘breakfast’^(22–24)^. The breakfast meal consisted of All-Bran Original cereal (Kellogg’s, Trafford, UK), semi-skimmed milk (Tesco, Welwyn Garden City, UK), Royal Gala apple (Tesco, Welwyn Garden City, UK) and orange juice (Tesco, Welwyn Garden City, UK), which provided 364 (sd 79) kcal (1524 (sd 331) kJ; i.e. 18 (sd 1) % of total daily energy needs) with 69 % carbohydrate, 13·5 % fat and 17·5 % protein of total meal energy. The calculated GI of the breakfast was 41 based on the International Tables of GI and Glycaemic Load values^(40,41)^ and the weighted means of the GI values for the component foods^(42)^. The provided breakfast contained 0·04 g of carbohydrate per kcal of individual measured RMR. A carbohydrate-rich breakfast was selected based on evidence that such meals can induce the second-meal effect^(14,15)^, with low GI meals successfully lowering glycaemic responses in subsequent meals^(43,44)^.

The inclusion of a high-fibre, low GI, cereal-based breakfast was designed to provide consistent metabolic responses across breakfast consumption conditions (EM-BC and MM-BC *v*. BO), ensuring that breakfast timing, the primary variable under investigation, could be examined without variability from meal composition. Additionally, this type of breakfast aligns with recommendations for promoting better cardiometabolic health in children and adolescents^(45,46)^. The portion size aligns with the recommendations that breakfast should contribute 15–25 % of daily energy needs^(22,23)^, and 55–75 % of energy should come from carbohydrates^(22)^. The timing of EM-BC aligns with the mean habitual breakfast timings of 07.39 (sd 00.49) on weekdays and 09.32 (sd 00.56) on weekend days reported by adolescent girls previously^(47)^. The timing of MM-BC challenges the proposed 10.00 cut-off for breakfast consumption and aligns with the typical school break time, enhancing ecological validity.

The lunch meal consisted of white bread without crust (Tesco, UK), margarine (Flora Buttery Spread, Tesco, UK), strawberry jam (Tesco, UK), salted crisps (Walkers, Reading, UK) and a glucose drink (Lucozade Energy Original, Coleford, UK), which provided 503 (sd 104) kcal (2105 (sd 436) kJ; i.e. 25 % of total daily energy needs) with ∼62 % carbohydrate, ∼33 % fat and ∼5 % protein of total meal energy. For the single vegan participant, vegan food options (Actileaf soya milk and Pure vegan sunflower spread) were provided. The provided lunch contained 0·05 g of carbohydrate per kcal of measured RMR, and the calculated GI was 73. The lunch was intentionally designed as a carbohydrate-rich, high-GI meal to challenge the glycaemic control of adolescent girls^(48)^ and enhance the likelihood of observing a second-meal effect. Both meals were consumed within 15 min. For participants who were unable to consume the entire breakfast or lunch, the quantities consumed in the first condition were replicated in subsequent conditions.

### Blood sampling and analysis

The whole hand was immersed in 40°C water for 5 min, followed by thorough drying, before a capillary blood sample was obtained by pricking the fingertip with a lancet (Unistick 3 Extra, Owen Mumford, UK). The first drop of blood was wiped, and 300–600 μl of blood was drawn into microvette tubes (Sarstedt Ltd., Leicester, UK). The sample tubes were placed immediately into a centrifuge at 12 800 g for 15 min (Eppendorf 5415c, Hamburg, Germany) to allow collection and storage of the resulting plasma at −80°C for subsequent batch analysis. Plasma glucose concentrations were determined by a benchtop analyser using enzymatic, colorimetric methods (Pentra 400; HORIBA ABX Diagnostics, Montpellier, France). Plasma insulin concentrations were determined using an enzyme-linked immunosorbent assay (Mercodia AB, Uppsala, Sweden). The intra-assay coefficient of variation was 0·6 % for plasma glucose and 3·7 % for plasma insulin. The homeostatic model assessment of insulin resistance (HOMA-IR, averaged across conditions) was calculated by dividing the result of multiplying fasting glucose and insulin by 22·5^(49)^. Participants ‘at risk’ of cardiometabolic disease were identified using age-, sex- and BMI-specific HOMA-IR percentiles^(50)^. Impaired fasting plasma glucose was determined according to the American Diabetes Association criteria (i.e. 5·6–6·9 mmol·l^−1^)^(51)^.

### Sample size estimation

A sample size of eleven adolescent girls was estimated to detect a statistically significant interaction (3 conditions × 2 block periods of pre- and post-lunch) in glucose incremental AUC (iAUC) at 80 % power with an *α* level of 0·05 and a Cohen’s *d* effect size of 1·47 based on a previous two-treatment crossover design study in adolescent girls^(16)^. At least fourteen participants were targeted for recruitment to allow for an anticipated 20 % dropout rate.

### Statistical analyses

Data were analysed using SPSS statistics software (version 25·0; SPSS Inc.). Postprandial outcomes were calculated for pre-lunch (breakfast manipulation period, time 0 through 4 h) and post-lunch period (from 4 through 6 h) using a time series response analyser^(52)^, which uses the trapezium rule. The total AUC (tAUC) and iAUC responses were divided by the respective time course to present the values in mmol·l^–1^ for glucose, pmol·l^–1^ for insulin and mg·min^–1^ for estimated substrate oxidation. For iAUC, areas below fasting levels were excluded. Additionally, a supplementary iAUC for delta glucose and insulin was calculated to account for baseline variability, reflecting relative postprandial changes. Linear mixed models were used to examine the differences between the three conditions in postprandial outcomes over two block periods of pre- and post-lunch. The linear mixed models included participants as a random effect and were adjusted for the order effect. Breakfast consumption frequency was considered as an additional covariate but was not included in the final analyses as it did not change the results significantly. Post-hoc pairwise comparisons were examined with the Holm–Bonferroni correction for multiple comparisons. The data residuals were checked for normality using Shapiro–Wilks tests. Normally distributed data are presented as mean (standard deviation (sd)). The skewed data of glucose tAUC and iAUC were naturally log-transformed before analyses. These data are presented as geometric mean (95 % CI), and analyses are based on ratios of geometric means and 95 % CI for ratios. Statistical significance was accepted at *P* < 0·05. Cohen’s effect size (*d*) was calculated to describe the absolute magnitude of difference according to the following thresholds: trivial (< 0·2), small (≥ 0·2 to < 0·5), moderate (≥ 0·5 to < 0·8) and large (≥ 0·8)^(53)^.

## Results

### Participant characteristics

Participant characteristics are presented in Table 1. One participant was classified as overweight and another one with obesity according to age and sex-specific BMI cut-off points^(28)^. Four girls, including those with overweight/obesity, were classified as ‘at risk’ according to age-, sex- and BMI-specific percentiles of HOMA-IR^(50)^. Two participants, including the one with overweight, had impaired fasting plasma glucose according to the American Diabetes Association criteria^(51)^. Four participants had not started their period.

**Table 1.** Participant characteristics (*n* 15) (Mean values and standard deviations; median values and interquartile ranges)

Variable	Mean		sd	Range
Age (years)	13·1		0·8	11·9–14·5
Stature (m)	1·56		0·68	1·43–1·68
Body mass (kg)	48·6		8·3	34·9–63·3
BMI (kg·m^–2^)	19·8		3·1	16·0–28·4
Body fat (%)	28·2		4·8	19·5–37·6
Lean body mass (kg)	34·6		4·5	28·1–43·1
Waist circumference (cm)	66·1		7·3	56·5–86·0
	Median	IQR		
Breast development^*^	4·0	1·0		2–5
Pubic hair development^*^	4·0	1·0		2–5
RMR (kcal·d^–1^)	1553		314	1060–2207

* Self-assessment – median (interquartile range).

### Breakfast habit questionnaire

Participants reported varying breakfast consumption frequencies: 4 d (*n* 3), 3 d (*n* 4), 2 d (*n* 2), 1 d (*n* 4) and never (*n* 2), resulting in an average of 2 (sd 1) days per week. The main reasons for skipping breakfast were ‘not feeling hungry’ (*n* 8) and ‘lack of time’ (*n* 5) on weekdays and ‘not feeling hungry’ (*n* 8) and ‘no motivation to prepare breakfast’ (*n* 4) on weekends.

### Meal composition

The average energy and macronutrient composition of breakfast and lunch meals consumed during the experimental conditions are presented in Table 2. The participants consumed, on average, 0·03 g carbohydrate per kcal of average RMR for breakfast, which is ∼0·01 g per kcal RMR less than the prescribed breakfast amount (i.e. 0·04 g carbohydrate per kcal RMR). This difference was most likely due to their infrequent breakfast consumption habit as some reported difficulty eating in the morning. Four participants consumed the prescribed breakfast quantity, while the rest (*n* 11) consumed at least 72 % of the prescribed breakfast. Seven participants consumed the full amount of the prescribed lunch (i.e. 0·05 g carbohydrate per kcal RMR), while the rest consumed at least 72 % of the prescribed lunch. Furthermore, participants who conducted the MM-BC first had a 5 % higher breakfast intake compared to those who started with the EM-BC condition. That said, all participants replicated the quantities consumed in the first condition in the subsequent conditions to minimise within-participant variability and thus isolate the possible effect of breakfast meal timing on the outcome variables.

**Table 2.** The average energy and macronutrient composition of breakfast and lunch meals consumed during the experimental conditions (*n* 15) (Mean values and standard deviations)

	Breakfast		Lunch	
	Mean	sd	Mean	sd
Energy (kJ)	1251	279	1917	412
Energy (kcal)	299	67	458	98
Carbohydrate (g)	52	12	71	15
Protein (g)	13	3	6	1
Fat (g)	4	1	17	4
Fibre (g)	14	3	3	1

### Plasma glucose responses

**Figure 3.**
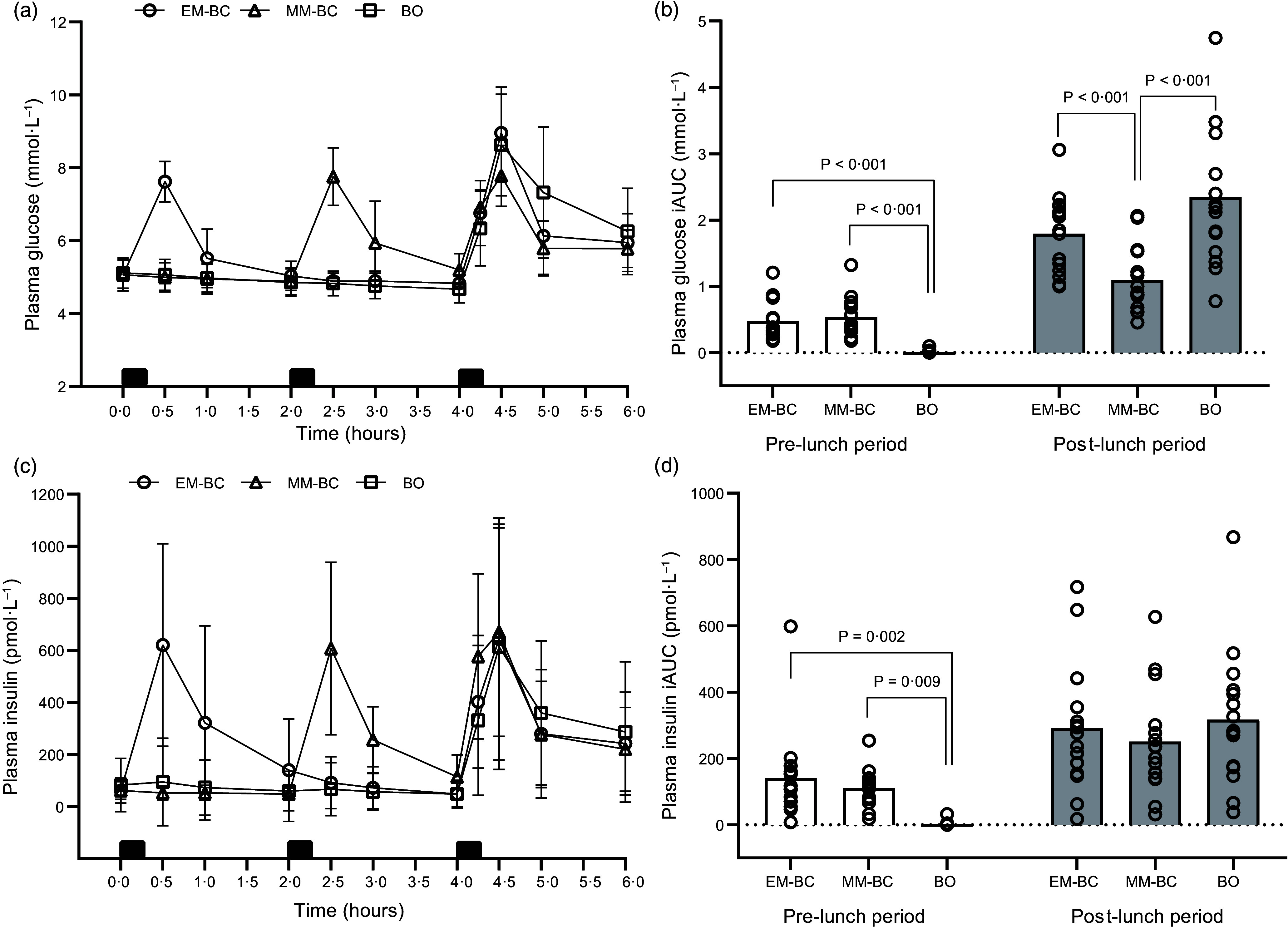
Changes in plasma glucose (a), glucose incremental AUC (iAUC) (b), plasma insulin (c), and insulin iAUC (d) concentrations during early-morning breakfast condition (EM-BC, open circle), mid-morning breakfast condition (MM-BC, open triangle) and breakfast omission (BO, open square). Data are presented as means and standard deviation. Black rectangles on panels (a) and (c) indicate consumption of breakfast (at 0 h for EM-BC and 2·0 h for MM-BC) and lunch meals (at 4·0 h for all conditions). Circles on panels (b) and (d) represent individual data values during the pre- (0–4 h, 240 min) and post-lunch periods (4–6 h, 120 min) of each condition. The iAUC (b) and (d) were divided by the time course of pre-lunch (240 min) and post-lunch periods (120 min) to present the values in mmol·l^–1^ for glucose and pmol·l^–1^ for insulin.

Plasma glucose concentrations over time and iAUC across the conditions are shown in Figure 3(a) and (b). Plasma glucose iAUC differed significantly between conditions (main effect: *P* = 0·033), with MM-BC showing a lower glucose iAUC than BO (–0·34 mmol·L^–1^; *P* = 0·033, *d* = 0·98) and EM-BC (–0·28 mmol·L^–1^; *P* = 0·123, *d* = 0·93), with no difference between EM-BC and BO (*P* = 1·00). Condition-by-time interactions were identified for glucose iAUC, tAUC and peak glucose (*P* < 0·001). During the pre-lunch period, both EM-BC and MM-BC resulted in significantly higher glucose iAUC, tAUC and peak glucose than BO (*P* ≤ 0·003, *d* = 1·31–5·03), with no differences between EM-BC and MM-BC (*P* ≥ 0·890, *d* = 0·28–0·41). During the post-lunch period, MM-BC reduced glucose iAUC, tAUC and peak glucose compared with BO (*P* ≤ 0·002, *d* = 0·68–1·44) and reduced iAUC and peak glucose compared with EM-BC (*P* ≤ 0·001, *d* = 0·74–0·92), with no significant differences in tAUC (*P* = 0·289, *d* = 0·32). Differences between EM-BC and BO post-lunch were moderate but non-significant for iAUC (*P* = 0·077, *d* = 0·52) and trivial to small for tAUC and peak glucose (*P* ≥ 0·195, *d* = 0·00–0·36). Mean glucose values for each condition and the mean differences between conditions during pre- and post-lunch periods are presented in Tables 3 and 4, respectively. Supplementary analysis of iAUC for delta glucose revealed similar results to those observed with raw glucose values. However, post-lunch iAUC for delta glucose for EM-BC became significant with a moderate effect compared with BO (*P* = 0·012, *d* = 0·55) as shown in online Supplementary Table S1.

**Table 3.** Mean glucose, insulin and substrates oxidation values for each condition and pairwise comparisons between conditions during pre-lunch period (0–4 h, 240 min) (Mean values and standard deviations; geometric mean, mean difference, percentage difference, and their corresponding 95% CI)

Outcome variables	Pre-lunch period (0–4 h, 240 min)						Pairwise comparisons								
	EM-BC		MM-BC		BO		EM-BC *v*. BO			MM-BC *v*. BO			MM-BC *v*. EM-BC		
	Geometric mean	95% CI	Geometric mean	95% CI	Geometric mean	95% CI	Percentage difference	95% CI	*d*	Percentage difference	95% CI	*d*	Percentage difference	95% CI	*d*
Glucose iAUC (mmol·L^–1^) ^†^	0·45	0·30, 0·61	0·52	0·37, 0·70	0·01	–0·09, 0·12	44 %^*^	21, 70	2·86	51 %^*^	27, 79	3·24	5 %	–11, 25	0·28
Glucose tAUC (mmol·L^–1^) ^†^	5·36	5·11, 5·61	5·52	5·27, 5·78	4·86	4·64, 5·09	10 %^*^	3, 18	1·31	14 %^*^	6, 22	1·72	3 %	–4, 10	0·41
	Mean	sd	Mean	sd	Mean	sd	Mean difference	95% CI	*d*	Mean difference	95% CI	*d*	Mean difference	95% CI	*d*
Peak glucose (mmol·L^–1^)	7·58	0·54	7·79	0·76	5·18	0·41	2·41^*^	1·73, 3·08	5·03	2·61^*^	1·94, 3·29	4·26	0·21	–0·47, 0·88	0·32
Insulin iAUC (pmol·L^–1^)	136	133	117	55	2	8	134^*^	42, 225	1·42	114^*^	23, 206	2·90	–19	–111, 72	0·19
Insulin tAUC (pmol·L^–1^)	192	178	174	77	68	90	124^*^	35, 213	1·38	106^*^	17, 195	1·18	–18	–106, 71	0·20
Peak insulin (pmol·L^–1^)	605	376	621	317	108	161	497^*^	335, 659	3·09	514^*^	352, 676	3·19	16	–146, 178	0·10
CHO oxidation tAUC (mg·min^–1^)	205	49	176	48	131	49	75^*^	43, 107	1·53	45^*^	13, 77	0·92	–29	–61, 2	0·59
Fat oxidation tAUC (mg·min^–1^)	36	12	46	19	55	12	–19^*^	–29, –8	1·58	–9	–19, 2	0·75	10^*^	0, 20	0·83

EM-BC, early-morning breakfast consumption; MM-BC, mid-morning breakfast consumption; BO, breakfast omission; *d*, Cohen’s effect size; iAUC, incremental AUC; tAUC, total AUC; CHO, carbohydrate.

For untransformed (normally distributed) data, values are expressed as mean (standard deviation (sd)) and pairwise comparisons are presented as mean absolute difference and corresponding 95 % CI.

The data were divided by the time course of pre-lunch period (0–4 h, 240 min) to present the values in mmol·l^–1^ for glucose, pmol·l^–1^ for insulin and mg·min^–1^ for estimated substrate oxidation.

* Indicates significant paired differences (*P* < 0·05). Mean differences are A compared with B (e.g. 10 % indicates EM-BC was higher on average than BO).

† For log-transformed data, values are presented as geometric means and corresponding 95 % CI, and pairwise comparisons are presented as percentage difference (%) based on ratios of geometric means and corresponding 95 % CI (%).

**Table 4.** Mean glucose, insulin and substrates oxidation values for each condition and pairwise comparisons between conditions during post-lunch period (4–6 h, 120 min) (Mean values and standard deviations; geometric mean, mean difference, percentage difference, and their corresponding 95% CI)

Outcome variables	Post-lunch period (4–6 h, 120 min)						Pairwise comparisons								
	EM-BC		MM-BC		BO		EM-BC *v*. BO			MM-BC *v*. BO			MM-BC *v*. EM-BC		
	Geometric mean	95% CI	Geometric mean	95% CI	Geometric mean	95% CI	Percentage difference	95% CI	*d*	Percentage difference	95% CI	*d*	Percentage difference	95% CI	*d*
Glucose iAUC (mmol·L^–1^)^†^	1·73	1·45, 2·04	1·05	0·85, 1·29	2·20	1·87, 2·56	–15 %	–28, 1	0·52	–36 %^*^	–46, –24	1·44	–25 %^*^	–37, –11	0·92
Glucose tAUC (mmol·L^–1^)^†^	6·57	6·28, 6·89	6·27	5·98, 6·56	6·93	6·62, 7·26	–5 %	–12, 2	0·36	–10 %^*^	–16, –3	0·68	–5 %	–11, 2	0·32
	Mean	sd	Mean	sd	Mean	sd	Mean difference	95% CI	*d*	Mean difference	95% CI	*d*	Mean difference	95% CI	*d*
Peak glucose (mmol·L^–1^)	8·92	1·22	7·89	0·70	8·91	1·39	0·002	–0·67, 0·68	0·00	–1·03^*^	–1·70, –0·35	0·74	–1·03^*^	–1·71, –0·36	0·74
Insulin iAUC (pmol·L^–1^)	286	187	257	155	317	199	–31	–123, 60	0·16	–61	–153, 31	0·31	–29	–121, 62	0·15
Insulin tAUC (pmol·L^–1^)	332	215	370	203	367	224	–35	–124, 54	0·16	3	–86, 92	0·01	38	–51, 127	0·17
Peak insulin (pmol·L^–1^)	636	448	721	374	683	455	–47	–209, 115	0·10	37	–125, 199	0·08	84	–78, 246	0·18
CHO oxidation tAUC (mg·min^–1^)	227	46	238	56	190	64	37^*^	5, 69	0·58	48^*^	17, 80	0·75	11	–20, 43	0·17
Fat oxidation tAUC (mg·min^–1^)	31	14	28	14	43	14	–12^*^	–22, –2	0·86	–14^*^	–25, –4	1·00	–2	–12, 8	0·14

EM-BC, early-morning breakfast consumption; MM-BC, mid-morning breakfast consumption; BO, breakfast omission; *d*, Cohen’s effect size; iAUC, incremental AUC; tAUC, total AUC; CHO, carbohydrate.

For untransformed (normally distributed) data, values are expressed as mean (standard deviation (sd)) and pairwise comparisons are presented as mean absolute difference and corresponding 95 % CI.

The data were divided by the time course of pre-lunch period (0–4 h, 240 min) to present the values in mmol·l^–1^ for glucose, pmol·l^–1^ for insulin and mg·min^–1^ for estimated substrate oxidation.

* Indicates significant paired differences (*P* < 0·05). Mean differences are A compared with B (e.g. –5 % indicates EM-BC was lower on average than BO).

† For log-transformed data, values are presented as geometric means and corresponding 95 % CI, and pairwise comparisons are presented as percentage difference (%) based on ratios of geometric means and corresponding 95 % CI (%).

### Plasma insulin responses

Plasma insulin concentrations over time and iAUC across the conditions are shown in Figure 3(c) and (d). Plasma insulin iAUC and tAUC showed no significant main effects for the condition (*P* ≥ 0·088). However, condition-by-time interactions were identified for insulin iAUC, tAUC and peak insulin (*P* ≤ 0·009). During the pre-lunch period, both EM-BC and MM-BC resulted in significantly higher insulin iAUC, tAUC, and peak insulin compared to BO (*P* ≤ 0·014, *d* = 1·18–3·19), with no differences between EM-BC and MM-BC (*P* = 1·00, *d* = 0·10–0·20). During the post-lunch period, differences in insulin iAUC, tAUC and peak insulin across conditions were trivial to small and non-significant (*P* ≥ 0·323, *d* = 0·01–0·31). Mean insulin values for each condition and the mean differences between conditions during pre- and post-lunch periods are presented in Tables 3 and 4, respectively. Supplementary analysis of iAUC for delta insulin revealed similar results to those observed with raw insulin values, as shown in online Supplementary Table S1.

### Fat and carbohydrate oxidation

**Figure 4.**
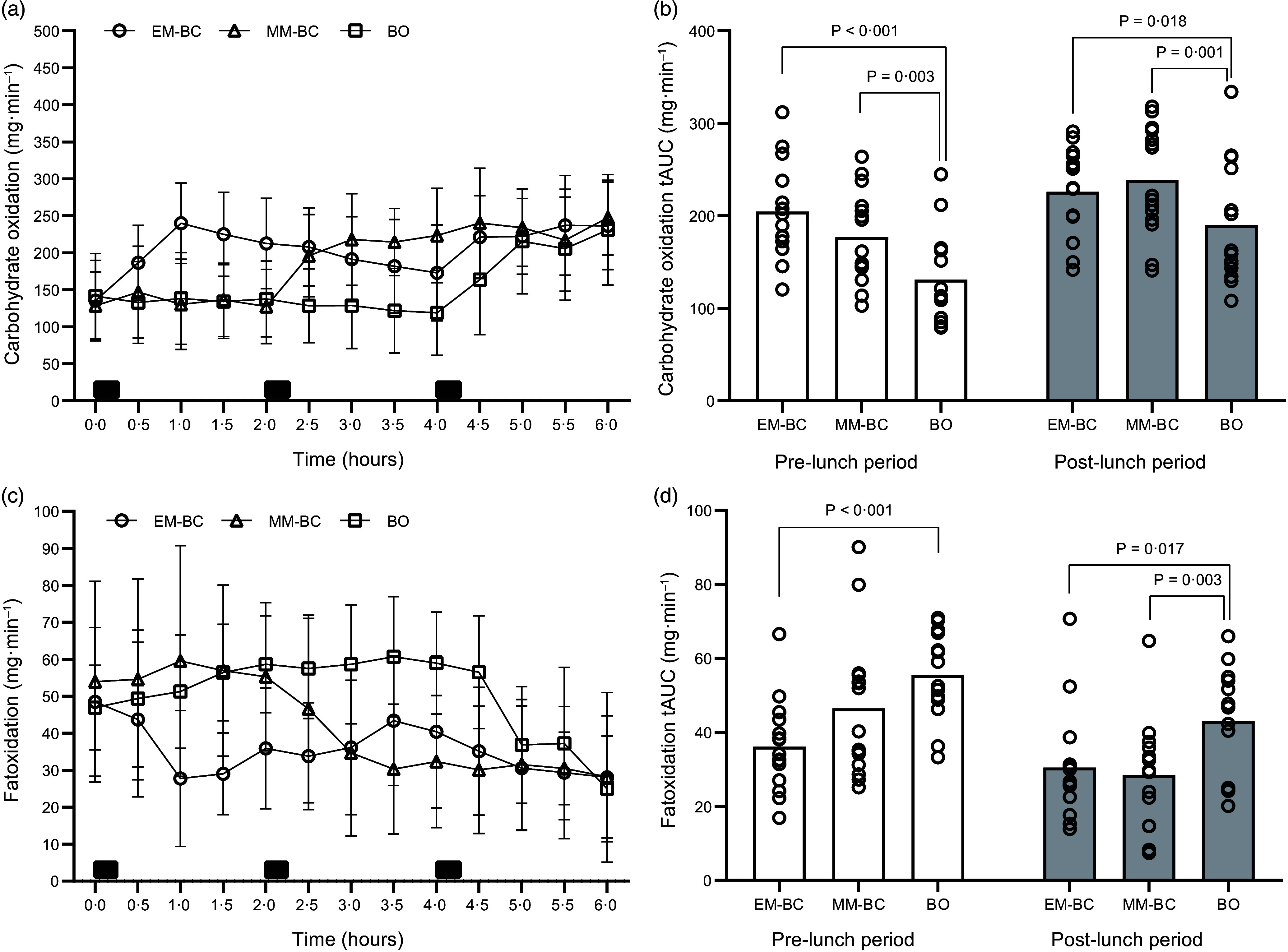
Changes in carbohydrate oxidation (a), carbohydrate oxidation total AUC (tAUC) (b), fat oxidation (c) and fat oxidation tAUC (d) during early-morning breakfast condition (EM-BC, open circle), mid-morning breakfast condition (MM-BC, open triangle) and breakfast omission (BO, open square). Data are presented as means and standard deviation. Black rectangles on panels (a) and (c) indicate consumption of breakfast (at 0 h for EM-BC and 2·0 h for MM-BC) and lunch meals (at 4·0 h for all conditions). Circles on panels (b) and (d) represent individual data values during the pre- (0–4 h, 240 min) and post-lunch periods (4–6 h, 120 min) of each condition. The tAUC (b) and (d) were divided by the time course of pre-lunch (240 min) and post-lunch periods (120 min) to present the values in mg·min^–1^.

Changes in substrate oxidation over time and tAUC across the conditions are shown in Figure 4. Substrate oxidation tAUC showed significant main effects for condition (*P* < 0·001). Both EM-BC and MM-BC resulted in higher carbohydrate oxidation tAUC (56 and 47 mg·min^–1^; *P* < 0·001) and lower fat oxidation tAUC (–15 and –11 mg·min^–1^; *P* ≤ 0·001) compared with BO, with no differences between the breakfast consumption conditions (*P* ≤ 0·569). Condition-by-time interactions were identified for carbohydrate oxidation (*P* = 0·047) but not for fat oxidation (*P* = 0·105). During the pre-lunch period, carbohydrate oxidation tAUC was significantly higher in EM-BC (*P* < 0·001, *d* = 1·53) and MM-BC (*P* = 0·003, *d* = 0·92) compared with BO, with a moderate reduction in MM-BC compared to EM-BC (*P* = 0·073, *d* = 0·59). During the post-lunch period, carbohydrate oxidation tAUC remained moderately higher in EM-BC and MM-BC compared with BO (*P* ≤ 0·018, *d* = 0·58–0·75), with no meaningful differences between EM-BC and MM-BC (*P* = 1·00, *d* = 0·17). Mean substrate oxidation values for each condition and the differences between conditions during pre- and post- lunch periods are presented in Tables 3 and 4, respectively.

## Discussion

This study examined the acute effect of breakfast timing (i.e. early morning and mid-morning) compared with BO on post-lunch glycaemia, insulinaemia and substrate oxidation in adolescent girls who infrequently consume breakfast. Both breakfast conditions reduced post-lunch glycaemic responses compared with BO, with MM-BC showing a stronger effect. These glycaemic improvements occurred without changes in post-lunch insulinaemia, suggesting an acute improvement in insulin sensitivity. Both breakfast conditions increased post-lunch carbohydrate oxidation compared with BO. However, only MM-BC reduced glycaemic responses when considering the combined effects of breakfast and lunch throughout the experimental period. These findings highlight the glycaemic benefits of consuming breakfast over skipping it, particularly in the mid-morning, in this population.

The reduction in glycaemic response after lunch with prior breakfast consumption *v*. BO has been referred to as the second-meal effect. This phenomenon has been reported previously in several studies in adults with^(14,54,55)^ and without T2D^(15,17,55,56)^, with fewer studies examining this effect in adolescents^(16,18)^. In one of these studies, the impact of consuming breakfast of varying protein content over 4 d on post-lunch glycaemic and insulinaemic responses was investigated in both breakfast skippers and consumers^(18)^. The breakfast skippers exclusively completed an additional BO condition, and the findings revealed that regardless of protein content, the inclusion of breakfast did not change glycaemic and insulinaemic responses to a lunch meal consumed 4 h later^(18)^. In contrast, the current study found a large reduction in post-lunch glucose iAUC with MM-BC *v*. BO, while EM-BC showed a moderate, non-significant reduction in post-lunch glucose iAUC (15 %). Supplementary analysis of delta glucose iAUC supported these findings and revealed a significant second-meal effect for EM-BC with a moderate effect size. The use of both raw and delta glucose iAUC in this study highlights the importance of considering different baseline adjustment approaches in metabolic research. While raw iAUC measures glucose responses above baseline, delta iAUC enhanced sensitivity by accounting for baseline variations. The differences in results between the current study and Alwattar et al.^(18)^ could be attributed to differences in participants’ characteristics such as age (mean 13 *v*. 19 years), weight status (mainly normal *v*. overweight/obese) and the breakfast habit (≤ 4 *v*. ≤ 2 d/week). Furthermore, while the energy and carbohydrate content of the breakfasts in the current study were more comparable to the normal-protein breakfast than to the high-protein breakfast provided by Alwattar et al.^(18)^, the GI of the breakfast meals were not specified, precluding direct comparisons.

Low GI meals are known to reduce immediate and subsequent (second meal) glycaemic responses^(43,44)^, impacting overall glycaemia. In this study, providing a low GI breakfast with a 2-hour time difference between the early-morning (08.30) and mid-morning (10.30) breakfast conditions did not result in distinct morning glycaemic and insulinaemic responses, indicating consistent insulin sensitivity^(57,58)^. However, a pronounced second-meal effect was observed with the shorter breakfast-to-lunch interval in MM-BC (2 h) compared with the longer interval in EM-BC (4 h) and BO (extended overnight fast), resulting in a lower glycaemic response over the experimental period. EM-BC showed a moderate second-meal effect compared with BO. These findings align with a recent study in habitual breakfast-consuming girls (≥ 4 d/week), which reported a large second-meal effect with a 3-h meal interval and a low GI breakfast compared with BO^(16)^. Both studies suggest that shorter intervals enhance the second-meal effect, despite differences in meal intervals and breakfast habits.

Another study in women with overweight/obesity using a 4-h meal interval found that breakfast consumption reduced post-lunch glycaemia compared with breakfast skipping, regardless of breakfast habit^(17)^. However, the negative impact of breakfast skipping on post-lunch insulinaemic response was only observed in habitual breakfast consumers (≥ 5 d/week), while habitual breakfast skippers (≤ 2 d/week) showed similar insulinaemic responses regardless of breakfast consumption, suggesting potential adaptive responses^(17)^. Similarly, in the current study of infrequent breakfast-consuming girls (≤ 4 d/week), breakfast consumption lowered post-lunch glycaemia compared with BO without meaningful changes in post-lunch insulinemia. Despite differences in participants’ characteristics and meal composition, these findings align with those of habitual skippers from the previous study^(17)^, suggesting that infrequent breakfast consumers may share similar metabolic adaptations. These results highlight the important role of breakfast consumption, timing and habitual patterns in optimising metabolic responses in this population.

A previous study in adults with T2D demonstrated that a high-protein, low-carbohydrate pre-load consumed 2 h before breakfast reduced postprandial glucose by 40 %^(59)^. In the current study, a similar effect was observed, with a 36 % reduction in post-lunch glucose iAUC when a breakfast meal was consumed 2 h before lunch but the effect was less pronounced when breakfast was consumed 4 h before lunch. The impact on postprandial glycaemia may vary based on prior meal composition and timing. More research is needed to determine the dietary (e.g. meal interval) and population-specific factors (e.g. breakfast habit) influencing the second-meal effect. Although the reduction achieved by MM-BC is smaller than the ∼45–50 % clinically relevant benchmark established for therapeutic interventions in individuals without diabetes^(60)^, this reference was included to highlight the magnitude of the observed reduction rather than for direct comparison. This novel finding highlights the potential benefits of subtle dietary adjustments in breakfast timing, with mid-morning breakfast offering a practical alternative for girls who infrequently consume breakfast, where early breakfast may be less common. Furthermore, the mid-morning breakfast in this study could be framed as a mid-morning ‘snack’ due to the timing of food intake. While there is no standardised definition of a ‘snack’^(61)^, this framing may align with adolescent eating habits and support practical recommendations for those who typically consume a mid-morning snack instead of breakfast. For these individuals, ensuring that a mid-morning snack is of sufficient quantity and quality could offer metabolic benefits similar to those observed in this study. If these responses are maintained over time, it could play a crucial role in moderating postprandial glycaemic responses and, thus, promote cardiometabolic health^(2)^ in those who infrequently consume breakfast.

The underlying mechanism of the second-meal effect has been linked to the suppression of NEFA concentrations and increased rate of muscle glycogen synthesis^(14,15)^. Elevated plasma NEFA concentrations demonstrate a dose-dependent inhibition of insulin-stimulated nonoxidative glucose disposal (through glycogen synthesis), with a lesser impact on muscle glucose oxidation^(62)^. Omitting breakfast elevates NEFA concentrations due to an extended fast^(15,63)^. However, consuming oral glucose reduces NEFA concentrations to a nadir within 2 h, and then rebounds above baseline values after 4 h in healthy adults^(64)^. Although not measured directly, it is likely that pre-lunch NEFA concentrations would be suppressed to a greater extent in the MM-BC than EM-BC condition compared with the BO condition. Therefore, the magnitude of the post-lunch glucose reduction was more pronounced in MM-BC than EM-BC when both were compared with BO, potentially influenced by the time interval between meals (i.e. 4 h in EM-BC and 2 h in MM-BC). Post-lunch carbohydrate oxidation was comparable between the breakfast consumption conditions, suggesting no significant differences in oxidative glucose utilisation. While measuring nonoxidative glucose disposal, including glycogen synthesis, would provide insight into the underlying mechanism, this is beyond the scope of the current study. Furthermore, such measurements in young people are limited due to invasiveness, posing practical and ethical challenges.

The strength of this study lies in its experimental design, which directly compared two breakfast timings against BO in infrequent breakfast-consuming girls. The purpose of the study was to acutely reduce postprandial glycaemia, as repeated reductions in postprandial glycaemia are known to help reduce cardiometabolic disease risk^(1,2)^, suggesting a beneficial daily practice. Conducted in a typically underrepresented group – apparently healthy, infrequent breakfast-consuming girls – our study addresses a gap in preventive health measures. However, the single-day crossover design limits insights into long-term adaptations, and the exclusion of mid-morning snacks may reduce generalisability to girls with habitual snacking patterns. While the design isolated the effects of breakfast timing without additional energy intake, future research should explore how mid-morning snacks influence metabolic outcomes. Furthermore, while our findings are promising, they are not directly generalisable to populations at higher risk (e.g. obesity and/or T2D); where the benefits of reducing postprandial glycaemia may be even greater. It is worth noting that there is no consensus on defining breakfast consumption frequency^(45)^, and consuming breakfast infrequently – even as often as four days a week – has been linked to increased cardiometabolic risk among adolescents^(33–35)^. Given the potential effects of habitual breakfast frequency on glucose metabolism^(17)^ and its variability among participants, sensitivity analyses incorporating breakfast consumption frequency as a covariate demonstrated that our findings remained consistent. While participants found the meals acceptable during the preliminary session, most girls (*n* 11) were unable to consume the entire breakfast, likely due to the timing of the meal and their infrequent breakfast consumption habits. Future research may benefit from considering smaller breakfast portions, including acclimatisation days, and assessing appetite and satiety to better understand the factors influencing meal consumption and metabolic responses in infrequent breakfast consumers. It should be emphasised that the breakfast portion remained standardised among participants and adhered to the recommended range of daily energy intake^(23)^. Although we initially controlled for the menstrual cycle phase (*n* 4), this was not possible across the whole sample due to the study recruitment plan of pairing the participants with friends to increase retention and enjoyment during the long testing sessions and other challenges such as the caregivers/participants commitments, irregular cycles and availability of research assistants and lab resources. Yet, studies based on adult women have been inconsistent regarding the effects of the menstrual cycle phase on insulin sensitivity and substrate oxidation at rest^(65,66)^.

In conclusion, the current study revealed that consuming breakfast lowers post-lunch glycaemic responses compared with skipping breakfast, with mid-morning breakfast demonstrating greater benefits over early-morning breakfast. These glycaemic improvements were achieved without significant changes in post-lunch insulinaemic responses and were accompanied by increased post-lunch carbohydrate oxidation. Importantly, mid-morning breakfast resulted in lower cumulative glycaemic responses across the breakfast and lunch periods. These findings underscore the importance of meal timing, specifically the between-meal interval, as a key determinant of glycaemic control in infrequent breakfast-consuming girls.

## Supporting information

Afeef et al. supplementary material Afeef et al. supplementary material

## Supplementary material

For supplementary material/s referred to in this article, please visit https://doi.org/10.1017/S0007114525000248
