# Supplementary material for: Impact of breakfast consumption timing v. breakfast omission on post-lunch glycaemia and insulinaemia in adolescent girls: a randomised crossover trial
Source: Br J Nutr. 2025 Feb 14;133(5):611–22. doi: 10.1017/S0007114525000248 (PMC12055447; doi:10.1017/S0007114525000248)
Supplement: Afeef et al. supplementary material [file S0007114525000248sup001.docx]

**Supplementary Table S1** Mean delta glucose and insulin incremental area under the curve (iAUC) for each condition and pairwise comparisons between conditions during pre-lunch (0–4 h, 240 min) and post-lunch (4–6 h, 120 min) periods

| **Outcome variables** | **Pre-lunch period (0–4 h, 240 min)** | | | **Pairwise comparisons** | | | | | | | |
| --- | --- | --- | --- | --- | --- | --- | --- | --- | --- | --- | --- |
|  | **EM-BC** | **MM-BC** | **BO** | **EM-BC vs BO** | | | **MM-BC vs BO** | | **MM-BC vs EM-BC** | | |
|  |  |  |  | **Mean difference**  **(95% CI)** | ***d*** | **Mean difference**  **(95% CI)** | | ***d*** | | **Mean difference**  **(95% CI)** | ***d*** |
| **Delta glucose iAUC (mmol·L^–1^)** | 0.46 ± 0.28 | 0.56 ± 0.28 | 0.01 ± 0.03 | 0.45  (–0.01 to 0.92) | 2.29 | **0.55***  **(0.09 to 1.02)** | | 2.73 | | 0.10  (–0.37 to 0.56) | 0.36 |
| **Delta insulin iAUC (pmol·L^–1^)** | 136 ± 133 | 117 ± 55 | 2 ± 8 | **134***  **(42 to 225)** | **1.42** | **114***  **(23 to 206)** | | **2.89** | | –19  (–111 to 72) | 0.19 |
|  | **Post-lunch period (4–6 h, 120 min)** | | | **Pairwise comparisons** | | | | | | | |
| **Outcome variables** | **EM-BC** | **MM-BC** | **BO** | **EM-BC vs BO** | | **MM-BC vs BO** | | | | **MM-BC vs EM-BC** | |
|  |  |  |  | **Mean difference**  **(95% CI)** | ***d*** | **Mean difference**  **(95% CI)** | | ***d*** | | **Mean difference**  **(95% CI)** | ***d*** |
| **Delta glucose iAUC (mmol·L^–1^)** | 1.78 ± 0.57 | 1.12 ± 0.48 | 2.34 ± 1.01 | **–0.56***  **(–1.03 to –0.10)** | **0.55** | **–1.22***  **(–1.69 to –0.76)** | | **1.21** | | **–0.66***  **(–1.13 to –0.20)** | **1.17** |
| **Delta insulin iAUC (pmol·L^–1^)** | 286 ± 187 | 257 ± 155 | 317 ± 199 | –31  (–123 to 60) | 0.16 | –60  (–153 to 31) | | 0.30 | | –29  (–121 to 62) | 0.16 |

EM-BC, early-morning breakfast consumption; MM-BC, mid-morning breakfast consumption; BO, breakfast omission; *d*, Cohen’s effect size; iAUC, incremental area under the curve.

* indicates significant paired differences (P < 0.05). Mean differences are A compared with B. (e.g., –0.56 indicates EM-BC was lower on average than BO).

Values are expressed as mean ± standard deviation (SD) and pairwise comparisons are presented as mean absolute difference and corresponding 95% CI.

The data were divided by the time course of pre-lunch period (0–4 h, 240 min) to present the values in mmol·L^–1^ for glucose, pmol·L^–1^ for insulin.
